# Glycophagy: molecular mechanisms, regulatory signals, and disease associations

**DOI:** 10.1080/27694127.2025.2595375

**Published:** 2026-01-08

**Authors:** Lei Chen, Jinyong Jiang, Meiqing Liu, Linxi Chen

**Affiliations:** a Institute of Pharmacy and Pharmacology, University of South China, Hengyang, China; b Department of Pharmacy, The First Affiliated Hospital of Jishou University, Jishou, China; c Yan’an Hospital Affiliated to Kunming Medical University, Central Laboratory, Kunming, China

**Keywords:** Glycophagy, STBD1, GABARAPL1, metabolic disorders, autophagy regulation

## Abstract

Glycophagy is a process of selective degradation of glycogen through the autophagy pathway. It relies on key proteins, such as STBD1 (glycogen-specific autophagy receptor), GABARAPL1 (member of the ATG8 family), and acid α-glucosidase (GAA), and proceeds through the steps of “glycogen recognition – autophagosome encapsulation – lysosomal degradation” to release glucose, thereby maintaining energy homeostasis. This process is regulated by multiple signaling pathways, such as AMPK, mTOR, CAMP/PKA, and calcium signaling pathways, which jointly respond to cellular energy demands and metabolic states. Glycophagy occurs under conditions, such as starvation, exercise, and energy metabolism disorders, and plays a role in diseases with glycogen metabolism disorders. Its functions include energy supply, blood sugar regulation, maintenance of cellular homeostasis, and influencing cellular aging. Dysfunction of glycophagy can lead to various diseases, such as glycogen storage diseases and diabetic cardiomyopathy. In-depth study of the regulatory mechanisms of glycophagy is helpful for developing therapeutic strategies for related diseases.

## Introduction

Glycophagy is a process of selective degradation of glycogen within cells through the autophagy pathway. It specifically degrades glycogen granules through the lysosomal pathway to meet energy demands or maintain metabolic homeostasis^[1–3]^. Studies have shown that the degradation of intracellular glycogen mainly involves two core pathways: the classical phosphorolysis pathway and glycophagy. The classical phosphorolysis pathway relies on enzyme systems such as glycogen phosphorylase and debranching enzymes, which break down glycogen in the cytoplasm into glucose-1-phosphate for cellular energy metabolism^[2]^. On the other hand, glycophagy is a selective autophagy process that targets glycogen granules to autophagosomes via the STBD1 autophagy receptor, followed by fusion with lysosomes^[2,4]^. Here, acidic α-glucosidase (GAA) degrades glycogen into free glucose, which is then released into the cytoplasm for energy utilization. These two pathways coexist in metabolically active tissues including the liver, skeletal muscle, heart, and brain. Due to differences in energy demands, each has evolved unique regulatory mechanisms and physiological functions. The classical phosphorolysis pathway relies on the rapid catalytic properties of its enzyme system and is mainly responsible for mobilizing glycogen quickly under short-term energy demands (e.g., acute exercise, short-term hunger). In contrast, glycophagy plays a supplementary or alternative role in specific physiological or pathological conditions (e.g., long-term energy stress and abnormal glycogen structure). Functional abnormalities in glycophagy (such as degradation disorders and regulatory imbalance) are closely linked to metabolic diseases including Pompe disease and diabetic cardiomyopathy. This further highlights its crucial role in maintaining cellular energy homeostasis and suggests that targeting glycophagy may represent a therapeutic strategy for related diseases.

It is worth noting that although the glycophagy pathway and the traditional phosphorylation pathway differ in terms of cellular localization, execution mechanisms, and kinetics, they are not completely independent at the regulatory level but rather exhibit synergy and complementarity. For instance, the key cellular energy sensor AMPK not only activates glycogen phagocytosis by phosphorylating ULK1 and STBD1 but also promotes the classical breakdown pathway by regulating glycogen phosphorylase^[4,5]^. Moreover, the specific receptor STBD1 of glycogen phagocytosis has been found to have potential interactions with key enzymes in glycogen metabolism. This multi-level cross-regulation ensures that cells can flexibly allocate the two degradation pathways according to the temporal and metabolic states of energy demand, thereby achieving the optimal utilization of glycogen reserves^[6–9]^.

## Occurrence of glycophagy

When cells encounter increased energy demands, such as during hunger, exercise, or energy metabolism disorders, they will activate glycophagy to break down glycogen and release glucose to provide energy to the cells. This helps maintain the normal functions of the cells and energy balance^[2,10]^. In pathological conditions, there are abnormalities in the synthesis or breakdown of glycogen, resulting in abnormal accumulation of glycogen within the cells^[11,12]^. To clear the abnormal glycogen particles, cells maintain the glycogen homeostasis within the cells through glycophagy. Cells will activate the autophagy pathway, including glycophagy, in response to various stress conditions, such as hypoxia, oxidative stress, endoplasmic reticulum stress, etc., to remove damaged organelles and abnormal proteins, maintaining the stability of the intracellular environment^[13]^. During cell apoptosis and necrosis, there is a display of glycogen accumulation within the cells, and the inhibition of glycogen metabolism can induce cancer cell death through the intrinsic apoptotic pathway^[14,15]^. Glycophagy may be involved in this process, helping cells break down and degrade glycogen and release cellular contents. In summary, the occurrence of glycophagy may be a comprehensive response of cells to energy demands, glycogen metabolism disorders, stress responses, and cell apoptosis.

### Key proteins of glycophagy

Glycophagy, as a specific autophagy pathway for cells to precisely degrade glycogen and maintain energy homeostasis, is initiated and executed through the cooperative action of core proteins. Among them, the glycogen-specific autophagy receptor protein STBD1 and the ATG8 family member GABARAPL1, respectively, play the key roles of “glycogen recognition and anchoring” and “autophagosome recruitment connection.” These two proteins form the core pathway for targeted glycogen degradation through specific interactions.

#### STBD1 (starch binding domain 1)

STBD1 (Starch Binding Domain 1) is a glycogen-specific autophagy receptor protein. It mediates glycophagy by recognizing glycogen and interacting with key autophagy factors^[6]^. Through its C-terminal CBM20 (Carbohydrate-Binding Module 20) domain, which forms a “dual-site binding pattern” by two independent oligosaccharide-binding sites, it recognizes the linear chain of α-1,4 glycosidic bonds and the α-1,6 branching points on the surface and in the deep cavity, achieving “dual-site binding” with glycogen and mediating the autophagy process. STBD1 is a peripheral membrane protein that is anchored to the endoplasmic reticulum (ER) and ER-mitochondria-associated membranes (MAMs) through its N-terminal hydrophobic segment^[16]^. The N-terminal hydrophobic segment is crucial for membrane anchoring: the absence of this domain results in the diffuse distribution of STBD1 in the cytoplasm^[9]^. The middle region of STBD1 contains the AIM motif, which includes key amino acids, such as W203, and specifically binds to the ATG8 family proteins, thereby anchoring the glycogen for delivery to lysosomes for degradation^[3,6,7]^. Furthermore, as the starting point of glycophagy, the function of STBD1 may also overlap with the classical glycogen breakdown pathway. Studies have shown that STBD1 can co-localize or interact with key proteins in glycogen metabolism, such as glycogen phosphorylase, suggesting its role as a bridge in the glycogen metabolism network^[8,9]^. When energy is abundant, the expression and activity of STBD1 are inhibited, and glycogen is mainly rapidly mobilized through the classical pathway. However, under long-term stress, STBD1-mediated glycophagy is activated, responsible for clearing glycogen with abnormal structures or those that cannot be effectively degraded by the phosphorylation pathway. This receptor-level regulation provides a new perspective for understanding how cells finely allocate resources for glycogen degradation.

#### GABARAPL 1 (glycophagy ATG8 partner protein)

GABA(A) receptor-associated protein 1 (GABA type a receptor-associated protein like 1, GABARAPL1) is one of the members of the ATG8 protein family and is involved in the formation and maturation of autophagosomes^[17,18]^. In glycophagy, the AIM of STBD1 (aa 203–206, W203-x-x-L206) selectively binds to mammalian ATG8 family members including GABARAPL1. Studies have shown that the aa 203–206 AIM on STBD1 interacts with GABARAPL1^[3,7,19]^. According to the current research, GABARAPL1 is currently the candidate “partner protein” of the ATG8 family in glycophagy with the most sufficient evidence. It associates with glycogen through STBD1 and promotes the formation of autophagosomes, thereby achieving the specific encapsulation of glycogen.

### Formation of autophagosomes

#### Recognition of glycogen and recruitment of autophagy-related proteins

STBD1 serves as a key autophagy receptor for glycophagy. It accomplishes inducing the formation of autophagosomes through the coordinated action of multiple domains. Among them, the CBM20 domain specifically recognizes the α-1,4 linear glucan chains and binds to the glucose residues at the α-1,6 branching points, thereby selectively recognizing glycogen^[6]^. The experiment has proved that mutating the carbohydrate-binding region significantly reduces the binding ability of STBD1 to carbohydrates (such as starch and glycogen)^[8]^. After the LC3 protein is synthesized, it is cleaved at its carboxyl terminus by ATG4, generating LC3-I localized in the cytoplasm. When the autophagy process is initiated, LC3-I forms a covalent bond with phosphatidylethanolamine (PE) through the ubiquitin-like modification system (dependent on ATG7 and ATG3) to form LC3-II (the membrane marker protein of the autophagosome)^[20,21]^. Finally, STBD1 binds to the ATG8-PE lipidated protein (GABARAPL1-PE) on the autophagosome membrane through its AIM domain, which is consistent with our previous research results. That is, the AIM of STBD1 can interact with the mammalian ATG8 family members, and GABARAPL1 is the most validated partner protein in glycophagy. This binding fixes glycogen granules on the surface of the phagophore, forming a glycogen-STBD1-GABARAPL1 ternary complex, thereby promoting the formation of glycogen autophagosomes^[6]^.

#### Formation and transport of autophagosomes

In glycophagy, the formation of autophagosomes begins with the nucleation of phagophores^[22]^. The initial stage of autophagosome formation relies on the activation of the ULK1 complex, which is activated by AMPK through phosphorylation of ULK1, thereby relieving the inhibition of mTORC1^[23]^. After ULK1 is activated, it may be indirectly localized to the glycogen-rich regions and collaborate with STBD1 to initiate nucleation. The CBM20 domain of STBD1 may target the VPS34-PI3K complex to the glycogen granules through dual functions (glycogen anchoring and Beclin1 recruitment), driving the generation of PI3P and the initiation of autophagosome membrane assembly. PI3P recruits proteins with WD40 domains (such as WIPI2), further promoting the extension and maturation of autophagosome membranes^[24,25]^. Through the helix–helix domain of ATG16L1, membrane curvature changes are induced, and the glycogen granules are encapsulated to form a closed vesicle structure^[26]^. The degradation of glycogen by glycophagy, after the formation of autophagosomes in the cytoplasm, is achieved through continuous directional transport mediated by microtubule motors (kinesin→dynein), enabling the fusion of autophagosomes and lysosomes^[27]^.

#### The degradation of glycogen in lysosomes

**Figure 1.**
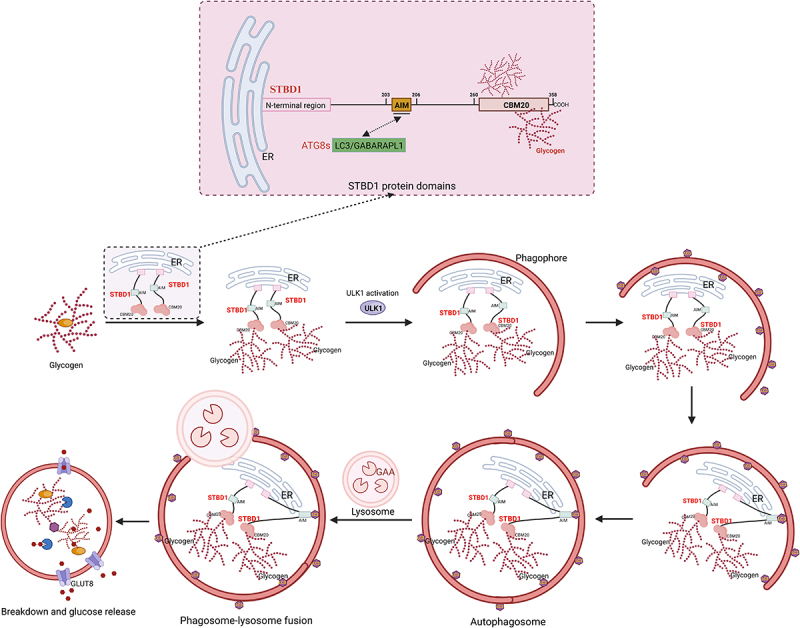
Diagram illustrating the structure of STBD1 and the process of glycophagy.

When autophagosomes are transported to the lysosomes, glycogen is degraded into glucose and released into the cytoplasm^[28]^. Acid α-glucosidase (GAA) is the key glycogen-degrading enzyme in the lysosome. Under acidic conditions, it hydrolyzes the α-1,4 and α-1,6 glycosidic bonds of glycogen, generating free glucose, which is transported to the cytoplasm through the glucose transporter on the lysosomal membrane (GLUT8 or G6PT)^[29]^. The cytoplasmic glucose can directly enter glycolysis, or when necessary, it is further mobilized by glycogen synthase to form cytoplasmic glycogen to generate glucose-1-phosphate, which is then converted into glucose-6-phosphate and enters the glycolysis pathway to produce ATP and NADH (Figure 1).

STBD1 specifically binds glycogen through the CBM20 domain and interacts with ATG8 family proteins (such as GABARAPL1) through the AIM (ATG8 interaction motif). Glycogen binds to STBD1 and is recruited to the autophagosome through the interaction with GABARAPL1. The mature autophagosome fuses with the lysosome, where GAA degrades glycogen into free glucose for metabolic recycling. STBD1: starch-binding domain protein 1, GABARAPL1: γ aminobutyric acid receptor-associated protein-like 1, GAA: acid α-glucosidase, and GlUT8: glucose transporter 8.

## Regulation of glycophagy

**Figure 2.**
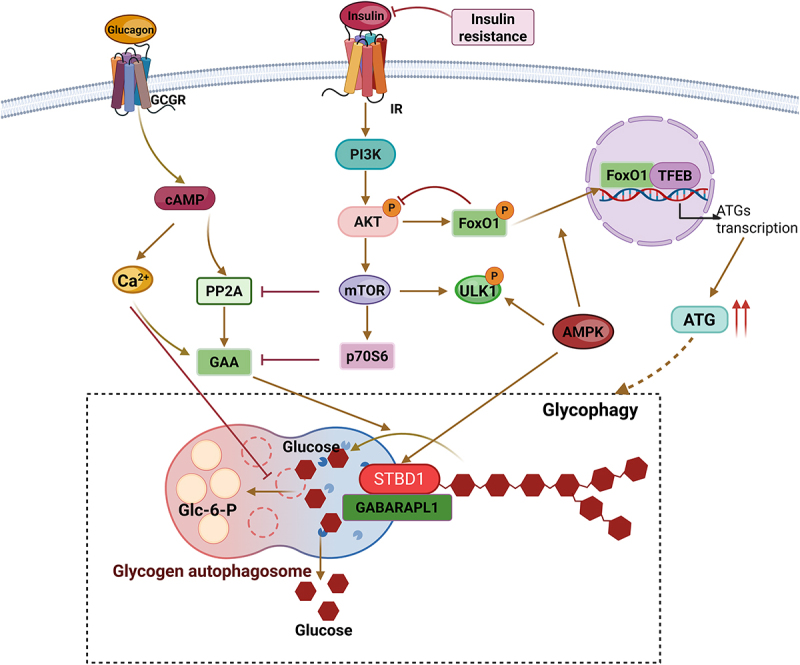
Regulation of glycophagy-related signaling pathways.

Glycophagy, as a key pathway for cells to respond to energy status and precisely regulate glycogen degradation, its initiation, execution, and dynamic balance are not the result of the action of a single factor. Instead, they rely on a multi-level collaborative network of signaling pathways, transcriptional regulation, post-transcriptional modifications, and hormone regulation. At the same time, it also has close interactions with cellular metabolic processes, such as glycolysis and oxidative stress. This regulatory network not only ensures that cells maintain a basal glycogen reserve when energy is abundant but also rapidly activates glycogen degradation when energy is stressed, providing energy for cells and maintaining blood sugar homeostasis (Figure 2).

Glycophagy is regulated by multiple signaling pathways, such as the AMP/protein kinase A, PI3K – Akt/PKB – mTOR, calcium pathway, as well as transcriptional regulation, post-transcriptional modification, hormones, such as insulin and glucagon, and also affected by ATP produced by glycolysis, HIF-1α, ROS, etc.

### Signaling pathway regulation

The initiation and dynamic regulation of glycophagy is highly dependent on the cell’s perception of its energy status. When cells are subjected to energy stress (such as insufficient energy supply or increased metabolic demands), multiple signaling pathways will synergistically activate or regulate the functions of autophagy-related proteins to ensure efficient degradation of glycogen and provide energy for the cells. Among them, the AMPK signaling pathway, the cAMP/PKA signaling pathway, and the calcium ion signaling are the core pathways that mediate the “energy status – glycophagy” regulation. They directly phosphorylate key proteins, regulate the balance of glycogen metabolism, and activate downstream feedback loops, jointly maintaining the precise execution of glycophagy.

#### AMPK signaling pathway

The AMP-activated protein kinase (AMPK) is a highly conserved protein that senses the cellular low-energy state and can detect changes in the ratio of AMP/ATP within the cell^[5]^. When the cellular energy level drops, the AMP level increases relatively, and AMP binds to the γ subunit of AMPK, causing a conformational change in AMPK and increasing its activity^[30,31]^. AMPK regulates the autophagy process through multiple mechanisms: On the one hand, it promotes autophagosome nucleation and membrane domain recruitment by phosphorylating the ULK1 complex and the VPS34 complex, directly accelerating autophagosome formation^[32]^. Phosphorylation of TSC2 and Raptor simultaneously inhibits the activity of mTORC1 (the high-energy sensor), relieving the inhibitory effect on autophagy initiation and activating the kinase activity of ULK1, forming a positive regulatory loop^[33,34]^. On the other hand, AMPK also indirectly affects autophagy by regulating the expression of autophagy-related genes through transcription factors, such as FOXO3 and TFEB, promoting the transcriptional expression of ATG genes, and constructing a transcriptional regulatory network for autophagy regulation^[35]^. In glycophagy, AMPK may enhance its glycogen-binding ability by phosphorylating STBD1, synergistically promoting the formation of glycogen autophagosomes, and by inhibiting mTORC1, relieving the inhibition of the ULK1 complex, thereby accelerating the maturation and degradation of GABARAPL1-mediated glycogen autophagosomes^[5,36]^.

Furthermore, the regulatory role of AMPK extends beyond glycophagy itself. It can also directly act on the traditional glycogen phosphorylation degradation pathway. AMPK can phosphorylate and activate glycogen phosphorylase kinase, thereby promoting the activation of glycogen phosphorylase and accelerating the cytoplasmic breakdown of glycogen^[37,38]^. Therefore, in the state of energy stress, the activation of AMPK constitutes a coordinated regulatory network: on the one hand, it achieves rapid mobilization of glycogen through the traditional pathway and on the other hand, it initiates the glycophagy pathway for long-term, selective glycogen clearance, and recovery. This dual-activation mechanism ensures that cells can maximize their efficiency in utilizing glycogen reserves to cope with energy crises.

#### The cAMP/PKA signaling pathway

The cAMP (cyclic adenosine monophosphate)/PKA (cAMP-dependent protein kinase) signaling pathway is also involved in the sensing of the energy state of glycophagy. Through the cAMP/PKA pathway, it activates PP2A, increasing the activity of glycogen hydrolase and providing more substrates for glycophagy. However, this step needs to be confirmed^[39]^. Under energy stress conditions, the intracellular cAMP level rises and activates PKA. PKA promotes glycogen breakdown by phosphorylating glycogen phosphorylase and inhibits glycogen synthesis by phosphorylating glycogen synthase^[40]^. Furthermore, cAMP increases the formation of autophagosomes and promotes the degradation of glycogen within these organelles, which is a response to the need for large amounts of glucose production^[41,42]^. This bidirectional regulation helps cells rapidly adjust the metabolic state of glycogen under energy stress conditions. Meanwhile, the cAMP/PKA signaling pathway has a synergistic effect with the AMPK signaling pathway. The increase in cAMP can activate AMPK, and the activation of AMPK further promotes the process of glycophagy, regulating autophagy through phosphorylation of key autophagy-related proteins and regulation of mTORC1 and ULK1 pathways^[43]^. Experimental findings show that cyclic AMP and cyclic AMP elevators (such as glucagon and epinephrine) stimulate glycogen phagocytosis in the liver, heart, and skeletal muscle, while cAMP antagonists (such as propranolol and exogenous glucose) lead to opposite changes^[44,45]^.

#### Calcium ion signal

Calcium ions (Ca^2+^) are crucial second messengers within cells, influencing both glycogen synthesis and breakdown, as well as initiating and progressing autophagy. Studies have shown that cAMP can alter the activity of calcium pumps and the possibility of opening calcium channels, thereby increasing cytoplasmic Ca^2+[46,47]^. In glycophagy, calcium ions can activate AMPK by activating calcium/calmodulin-dependent protein kinase (CaMKKβ), forming a calcium-AMPK positive feedback loop to promote glycophagy^[40,48]^. In vitro experiments suggest that an increase in cytoplasmic Ca^2+^ can enhance the hydrolytic activity of GAA on glycogen, accelerating the autophagy process^[49]^. In lysosomes, TRPML1-Ca^2+^ signals regulate the fusion of lysosomes and autophagosomes, ensuring that glycogen particles can be effectively encapsulated and degraded during the process of glycophagy^[50–52]^.

### Transcriptional regulation

When there is long-term hunger, oxidative stress, or weakened insulin signaling, FoxO1 enters the nucleus through dephosphorylation. FoxO-mediated transcriptional reprogramming (such as upregulation of gluconeogenesis, lipolysis, and autophagy-related genes) ensures the continuous energy supply during long-term starvation. When insulin levels are low, FoxO1 can enter the nucleus and bind to the GABARAPL1 promoter, promoting its transcription, thereby promoting glycophagy^[53]^. During energy stress (AMPK activation) or when lysosomal function is impaired, TFEB translocates from the cytoplasm to the nucleus through mTORC1 inactivation, enhancing lysosomal generation and acidification capabilities and promoting glycogen recognition and degradation capabilities^[54]^. TFEB works in synergy with FoxO to jointly activate genes such as STBD1 through chromatin remodeling complexes^[55]^. At the same time, the Ca^2+^/CaM signaling pathway activates TFEB through CaMKK2 and AMPK, promoting its nuclear translocation and upregulating the expression of genes related to autophagy, thereby enhancing autophagy efficiency^[56]^.

### Posttranslational modification

Studies have shown that the N-terminal region of STBD1 can be ubiquitinated and marked, leading to its degradation by the proteasome, thereby regulating its expression level within the cell^[8]^. The dynamic balance of ubiquitination modification regulates the expression level of STBD1, ensuring the maintenance of basal autophagy when glycogen is abundant and enhancing the stability of STBD1 in stress states, such as nutrient deficiency by inhibiting ubiquitination and promoting glycogen degradation. Additionally, Ducommun et al. discovered through chemical genetic screening that the Ser175 site of the STBD1 protein can be phosphorylated by AMPK in various cells and tissues, suggesting that STBD1 is a new substrate of AMPK and may be involved in regulating cellular processes related to glycogen metabolism^[57]^. Glycophagy is regulated by the AMPK signal, and the AMPK-dependent phosphorylation of STBD1 may be a key link connecting energy sensing, autophagy, and glycogen metabolism.

Other post-translational modifications (such as acetylation^[58]^ and glycosylation) exist in glycophagy and other autophagy processes. The current research has not fully clarified these phenomena, and they deserve further exploration.

### Hormone regulation

#### Insulin

Insulin binds to the insulin receptors on the surface of target cells and activates downstream signaling pathways. Among them, the PI3K-Akt-mTORC1 pathway is one of the most crucial signaling pathways. Insulin binding to the receptor activates the PI3K-Akt pathway, where Akt is phosphorylated and inhibits AMPK while activating mTORC1, which phosphorylates ULK1 and TFEB, inhibiting the formation of autophagosomes and the generation of lysosomes^[59]^. At the same time, insulin can activate Akt, thereby inhibiting the binding of FoxO to the GABARAPL1 promoter and inhibiting the transcription of GABARAPL1, thereby inhibiting the entry of glycogen into lysosomes and reducing glycophagy to lower blood sugar levels^[53,60]^.

#### Glucagon

Glucagon is secreted when blood glucose levels are low and it can induce glycophagy by activating the cAMP-PKA pathway. Glucagon activates the receptor on the liver cell membrane, elevates the cAMP level, activates PKA, and thereby phosphorylates AMPK, enhancing its activity, and subsequently activates ULK1 and STBD1 to initiate glycophagy^[41]^. Glucagon also inhibits mTOR by regulating glycogen hydrolase and autophagic activity^[59,61]^. Additionally, it synergistically increases intracellular Ca^2+^ concentration through calcium signaling to affect GAA activity^[61]^.

### Other

The catabolism of glycogen mainly involves two distinct pathways: the glycolytic process and the lysosome-dependent glycophagy process. The ATP produced during the glycolytic process can inhibit the activation of mTOR kinase (as the core molecule for cellular energy sensing), thereby suppressing the activity of the autophagy initiation complex (such as ULK1-ATG13), and thus reducing the occurrence of autophagy. At the same time, HIF-1α is activated under hypoxia or energy stress and can induce autophagy by transcriptionally upregulating autophagy-related genes. This autophagy activation can, in turn, affect the switching of glycolytic metabolism^[62]^. Meanwhile, autophagy is crucial for maintaining redox homeostasis as it eliminates dysfunctional macromolecules and organelles, thereby reducing oxidative damage and promoting cell survival^[63]^. Reactive oxygen species (ROS) act as cellular stress signals (such as oxidative stress and energy deprivation) and can activate AMPK either directly or indirectly; for example, ROS can oxidize the cysteine residues of AMPK or inhibit its phosphatase activity, promoting the phosphorylation and activation of AMPK. Activated AMPK, on the one hand, phosphorylates and inhibits mTOR (the negative regulator of autophagy), and on the other hand, directly phosphorylates ULK1 (the core kinase of the autophagy initiation complex), releasing the inhibitory binding of ULK1 to mTOR and enhancing its kinase activity, thereby initiating autophagy^[5]^. Therefore, the activation of autophagy induced by ROS through the AMPK-ULK1 pathway theoretically accelerates the lysosome-dependent degradation of glycogen, providing glucose for the cell to meet the energy demands under stress conditions^[64]^.

In conclusion, glycophagy is not only a “backup pathway” for energy metabolism but also a complex regulatory node, integrating hormones, oxidative stress, calcium signaling, epigenetics, and mechanical microenvironment. Its dysregulation is closely related to metabolic diseases and neurodegenerative disorders. In the future, it is worthy of in-depth exploration from the perspectives of organelle interactions, single-cell metabolomics, and drug intervention.

## The role of glycophagy in different cells

Glycophagy plays a central regulatory role in various diseases through multiple signaling pathways (such as PI3K/AKT, AMPK, and FOXO1): it has protective effects in myocardial ischemia and ovarian aging but may cause dysfunction due to pathway inhibition in high-carbohydrate diets and ischemic stroke. Its synergistic effect with mitochondrial autophagy provides potential therapeutic targets for metabolic diseases and neurodegenerative disorders.

**Figure 3.**
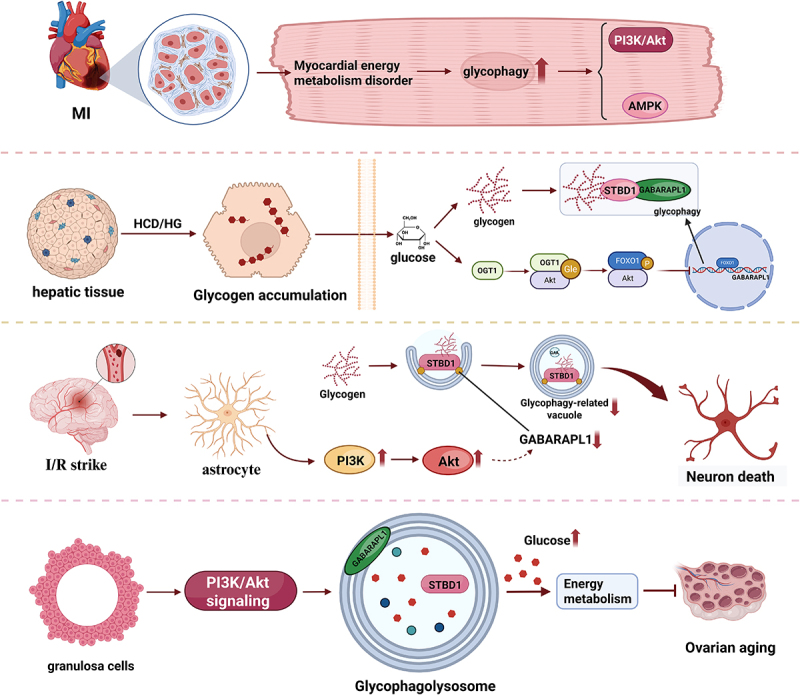
Glycophagy participates in the functional regulation of different cells.

Studies have shown that in the case of oxygen and nutrient supply interruption caused by myocardial ischemia, glycophagy is considered to be part of the protective mechanism of the myocardium in ischemic injury. Research has found that by regulating glycogen metabolism, glycophagy can alleviate the energy stress of myocardial cells and reduce cell damage^[65–67]^. Meanwhile, the reactive oxygen species (ROS) produced by the damaged mitochondria during ischemia further activate the signaling pathways related to glycophagy. Glycophagy, together with mitochondrial autophagy (mitophagy), jointly regulates the energy metabolism of cardiac cells and provides protection for the heart^[68]^. In a high-carbohydrate diet and high-glucose environment, the accumulation of glycogen in liver tissue and liver cells will induce the expression of AKT1, and through the OGT1-AKT1-FOXO1 pathway, they inhibit glycogen phagocytosis and glucose production^[69]^. By inhibiting FOXO1 from entering the cell nucleus and reducing its recruitment to the GABARAPL1 promoter, the activity of glycophagy is reduced. In ischemic stroke, chronic hypoxia/glucose deprivation leads to insufficient brain energy supply. Studies have found that the dysfunction of glycophagy in astrocytes is related to the downregulation of GABARAPL1, and this downregulation is driven by the PI3K-Akt pathway during cerebral reperfusion. The deficiency of glycophagy leads to a lack of amino glucose, which inhibits the nuclear translocation of specific protein 1 and TATA-binding protein, thereby feedback-inhibiting GABARAPL1-mediated neuronal death in astrocytes^[70]^. Additionally, during ovarian aging, follicle-stimulating hormone (FSH) can promote glycophagy by activating the PI3K/AKT signaling pathway, thereby increasing glycogen utilization and providing energy support for senescent granulosa cells (GCs)^[71]^. It should be noted that this phenomenon of FSH-mediated glycophagy activation, which is dependent on PI3K/AKT, was observed in non-mammalian models (chickens). This might represent a cell-type-specific regulatory mechanism. This is different from the typical role of the insulin-activated PI3K/AKT pathway in inhibiting glycophagy in metabolic tissues, such as the liver and muscle. At the same time, glycophagy promotes mitophagy through activating the AMPK signaling pathway, removes damaged mitochondria, reduces ROS production, and protects granular cells from oxidative stress damage (Figure 3).

In various physiological and pathological conditions, such as myocardial ischemia, high-carbohydrate diet, ischemic stroke, and ovarian aging, glycophagy exerts protective effects on cells and regulates energy through regulating related signaling pathways (such as PI3K-Akt, AMPK, etc.) and collaborating with other autophagy processes (such as mitochondrial autophagy).

## The function of glycophagy

**Figure 4.**
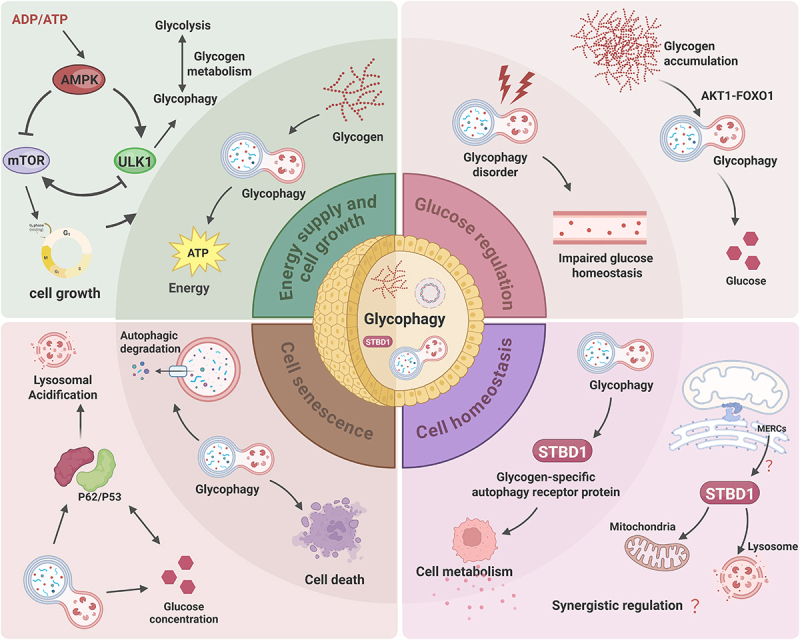
Glycophagy dynamically regulates glycogen degradation.

Glycophagy, as a special form of cellular autophagy, participates in multiple physiological processes by selectively degrading glycogen granules. Its core functions can be summarized as follows (Figure 4).

Glycophagy dynamically regulates glycogen degradation. It plays a crucial role in maintaining energy homeostasis (during starvation/tumor proliferation), regulating blood sugar (as a therapeutic target for diabetes), and in cellular senescence (due to the decline of lysosomal function). Its function depends on the formation of autophagosomes, the collaboration of organelles at the mitochondria-endoplasmic reticulum contact sites (MERCs), and the regulation of metabolic balance by the STBD1 protein.

### Energy supply and cell growth

When the body is hungry or lacks energy, cells break down the stored glycogen through glycophagy to generate glucose for energy supply, maintaining the basic metabolic requirements^[72]^. Glycophagy, through lysosomal degradation of glycogen, serves as an important energy metabolism pathway, operating concurrently with the traditional glycogen breakdown pathway^[2]^. Regarding glycophagy, studies have found that it can promote cell growth and proliferation by providing energy and metabolic intermediates and can also inhibit excessive cell growth and proliferation by regulating cellular metabolic balance and cell cycle^[73,74]^. Glycophagy provides glucose and pyruvate to support the progression of the cell cycle and cell proliferation. This regulatory mechanism ensures that cells can flexibly adjust their metabolic activities according to the nutritional status, supporting cell growth and survival^[75]^. In a high-nutrition state, mTORC1 promotes protein synthesis and cell growth by phosphorylating S6K and 4E-BP1, while inhibiting glycophagy^[76,77]^. Conversely, in a state of energy deficiency, AMPK is activated and inhibits mTORC1, initiating glycophagy, and inhibiting the expression of cell cycle proteins through ULK1 phosphorylation, resulting in G1 phase arrest^[78–80]^. Studies have also found that in some tumor cells, the activity of glycophagy is abnormally increased, providing energy and metabolic substrates for tumor cells, promoting their rapid growth and proliferation^[81]^.

### Blood glucose regulation

Studies have shown that glycophagy may be abnormal in diabetes models, affecting glycogen metabolism and blood glucose homeostasis^[82]^. Glycophagy regulates blood glucose through the AMPK and mTOR signaling axes: AMPK is activated in the absence of glucose and initiates autophagy by phosphorylating ULK1 and promotes the nuclear localization of FOXO1 to enhance the transcription of GABARAPL1 and STBD1, thereby activating glycophagy (energy production) and inhibiting glycogen synthesis (energy consumption)^[33,83]^. While mTOR regulates cellular metabolic activities by sensing glucose levels and affects the regulation of blood glucose^[84,85]^. At the same time, under energy stress conditions, glycophagy is activated to accelerate the degradation of liver glycogen, thereby providing energy^[86]^. Studies have shown that high blood sugar or high carbohydrate intake can inhibit glycophagy through signaling pathways such as AKT1-FOXO1, reduce glucose release, and prevent excessive blood sugar levels^[69]^. Glycophagy may become a new target for the treatment of diabetes and its complications. By regulating the autophagy pathway, it is possible to improve glycogen metabolism and blood sugar homeostasis^[6,87]^.

Research has shown that glycophagy may become a new target for the treatment of diabetes and its complications. By regulating the autophagy pathway, it is possible to improve glycogen metabolism and blood glucose homeostasis.

### Cell homeostasis maintenance

Glycophagy maintains cellular metabolic homeostasis by selectively eliminating damaged or excessive glycogen granules. Its dysfunction can lead to abnormal glycogen deposition and cause pathological changes, such as the accumulation of abnormal glycogen in Lafora disease, which is closely related to the deficiency of glycophagy^[88]^. As a key protein of glycophagy, STBD1 not only mediates the targeted binding of glycogen to autophagosomes but also localizes at the mitochondrial-endoplasmic reticulum contact sites (MERCs). By regulating the formation of endoplasmic reticulum structure, mitochondrial-endoplasmic reticulum binding, and mitochondrial morphology, it participates in the coordinated regulation of cellular energy metabolism and autophagy initiation^[16,89]^. This trans-cellular localization suggests that glycophagy may collaborate with the autophagosome-lysosome system and other organelles, such as mitochondria, to optimize the distribution and utilization of energy substrates, providing a new perspective of cross-cellular regulatory mechanism for understanding its role in metabolic homeostasis and diseases.

### Cell aging

Autophagy inhibits cellular aging by degrading damaged organelles and macromolecules, but some studies have also shown that autophagy can promote cellular aging by facilitating the synthesis of senescence-associated secretory phenotypes^[90–92]^. Research has found that the lifespan of yeast cells can be extended by periodically changing the concentration of glucose in the environment, indicating a close relationship between glycogen metabolism and cellular lifespan^[93,94]^. As cells age, the function of autophagy gradually declines, and the decreased activity of lysosomal enzymes leads to disordered glycogen metabolism within the cells, abnormal accumulation, or decomposition of glycogen, which further affects the energy metabolism and functions of the cells, accelerating cellular aging^[95,96]^.

## Glycophagy and disease

**Figure 5.**
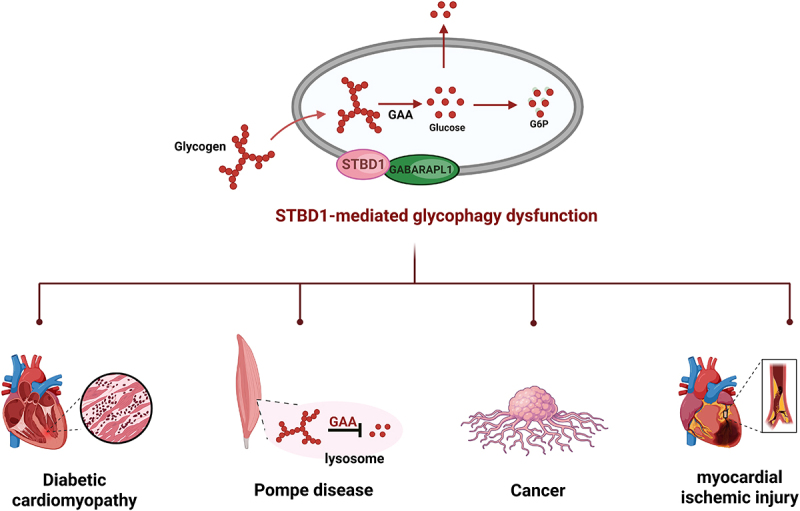
Dysfunction of glycophagy mediated by STBD1 in human diseases.

Glycophagy, as the core pathway for cells to precisely regulate glycogen degradation and maintain energy homeostasis, its dysfunction, or imbalance in regulation will directly lead to disorders in glycogen metabolism and thereby participate in the occurrence and development of various diseases. From hereditary glycogen storage diseases to metabolic disorders, to tumors, liver diseases, etc., the abnormality of glycophagy plays a key pathological role^[97,98]^ (Figure 5).

The dysregulation of the glycophagy process mediated by STBD1 can lead to the occurrence of the following diseases and disorders. For instance, glycogen storage disease, diabetic cardiomyopathy, tumors, and nonalcoholic fatty liver disease, etc. Its role in diseases is complex and is closely related to multiple signaling pathways and molecular mechanisms.

### Glycogen storage diseases (GSDs)

The abnormal function of glycophagy is mainly dominated by the direct effect of blocked glycogen degradation. Through the functions of enzymes related to the autophagy pathway (such as acid α-glucosidase GAA), the deficiency of these enzymes directly hinders the degradation of glycogen in lysosomes. Pompe disease (PD), as a type II autosomal recessive glycogen storage disorder, its core cause is the mutation of the gene encoding lysosomal GAA, which leads to the deficiency of GAA, blocking the degradation steps of glycogen by lysosomes during the process of autophagy (rather than the fusion disorder of autophagosomes and lysosomes), ultimately resulting in abnormal accumulation of glycogen in lysosomes^[99–102]^. The only specific treatment for Pompe disease is enzyme replacement therapy (ERT), which aims to restore the efficiency of lysosomal glycogen degradation by supplementing the deficient GAA enzyme, thereby slowing down the disease progression and prolonging the lifespan of infants with the condition^[103]^. Studies have found that the inhibitory effect of mTORC1 on glycogen accumulation in Pompe disease muscle may be complex, and the expression level of STBD1 in the skeletal muscle of PD mice is highly elevated, suggesting that glycophagy may alleviate glycogen accumulation in lysosomes through compensatory mechanisms and play a protective role^[104,105]^. Another related disease, Lafora disease, is caused by the loss of function of the laforin or malin genes, resulting in abnormal glycogen chain structure (reduced branching, increased chain length), forming Lafora bodies that are difficult to degrade and accompanied by abnormal autophagy regulation^[106–109]^. Glycophagy dysfunction is considered to be an important pathological mechanism of this disease, and overexpression of STBD1 may improve the clearance of abnormal glycogen and improve the disease phenotype^[88]^. In summary, the functional defect or regulatory abnormality of glycophagy is an important pathological mechanism of glycogen storage diseases, and targeting the enhancement of its clearance function may provide a new direction for the treatment of related diseases.

### Diabetic cardiomyopathy (DCM)

In diabetic cardiomyopathy, the disorder of the myocardial cell glycophagy pathway manifests as impaired formation of autophagosomes or defects in lysosomal degradation, resulting in abnormal accumulation of glycogen in the cytoplasm or lysosomes and abnormal subcellular localization. This abnormality directly reduces the effective degradation of glucose and ATP production, disrupts the energy metabolism homeostasis of the myocardium, induces oxidative stress and mitochondrial dysfunction, and ultimately leads to decreased myocardial contractility, myocardial hypertrophy, and fibrosis, etc., which are structural remodeling^[110–112]^. In diabetic cardiomyopathy, the accumulation of glycogen within myocardial cells and the disorder of glycophagy flux are among its characteristics^[110,113,114]^. The pathological phenomenon of increased glycogen deposition in diabetic myocardium is evident in both humans and a series of experimental models^[115,116]^. Studies have found that in diabetic cardiomyopathy, insulin deficiency/resistance leads to myocardial glycogen deposition and compensatory activation of glycophagy^[66,113]^. It is notable that different characteristics are exhibited in type 1 and type 2 diabetes: in type 1 diabetes, autophagy activity is enhanced, while in type 2 diabetes, the autophagy flow is interrupted due to the blocked lysosomal degradation steps^[117,118]^. Interestingly, the role of glycophagy in cardiac metabolic stress may vary by gender; in female hearts, there is a higher glycophagy activity under fasting conditions^[117,119]^. In diabetic cardiomyopathy, the upregulation of glycophagy may reduce the sensitivity of myocardial cells to insulin, thereby exacerbating myocardial damage^[53,120,121]^. Mechanistically, endogenous FoxO1 is dephosphorylated and activated in the nucleus and then directly binds to the promoter region of GABARAPL1 under glucose deprivation conditions, promoting subsequent myocardial cell transcription^[53]^. Therefore, it is hypothesized that FoxO1, as an important mediator of diabetic cardiomyopathy, may become an attractive therapeutic strategy.

Diabetes is a chronic metabolic disease caused by insulin resistance or deficiency, which leads to elevated plasma glucose levels. One of the characteristics of diabetic cardiomyopathy is the accumulation of glycogen in cardiomyocytes and the disorder of glycophagy flux^[110,113,114]^. The pathological phenomenon of increased glycogen deposition in diabetic cardiomyocytes is obvious in both humans and a series of experimental models^[115,116]^. Studies have found that autophagy of glycogen in cardiomyocytes is affected by insulin and high glucose environment under diabetic conditions, resulting in increased glycogen accumulation and increased expression of glycophagy markers STBD1 and GABARAPL1^[66,113]^. The regulation of autophagy in diabetic cardiomyopathy shows different characteristics in type 1 and type 2 diabetes: autophagy activity is enhanced in type 1 diabetes, while the autophagy process is inhibited in the final digestion step in type 2 diabetes^[117,118]^. Interestingly, the role of glycophagy in cardiac metabolic stress may vary by gender, with higher glycophagy activity in female hearts under fasting conditions^[117,119]^. In diabetic cardiomyopathy, the upregulation of glycophagy may reduce the sensitivity of cardiomyocytes to insulin and aggravate myocardial injury^[53,120,121]^. Endogenous FoxO1 is dephosphorylated and activated in the nucleus and then directly binds to the promoter region of GABARAPL1 under glucose deprivation conditions, promoting subsequent myocardial cell transcription^[53]^. Therefore, it is speculated that FoxO1 is involved in glycophagy as an important mediator in diabetic cardiomyopathy, and it may become an attractive therapeutic strategy.

### Other

Glycophagy plays a complex and dual role in the development of tumors. Initially, the research results regarding its impact on tumor growth were contradictory; however, these differences reflect the different functional characteristics it exhibits due to the unique environment of the tumor. On the one hand, the absence or impairment of glycophagy can drive tumor occurrence. The dysfunction of the glycogen-specific autophagy receptor STBD1 leads to glycogen accumulation and inhibition of glycophagy, which has been proven to promote metabolic reprogramming and tumor growth, suggesting that the impairment of glycophagy is closely related to tumor occurrence^[19]^. Similarly, STBD1 is significantly downregulated in cancers such as colon cancer, which directly impairs the targeting of glycogen to autophagosomes and may promote the growth and metabolic adaptation of tumor cells. Moreover, in tumor cells, the reduction of methylation of key transcription factors (FoxO and TFEB) decreases autophagy activity, further exacerbates the dysfunction of sugar uptake, and promotes tumor growth^[122,123]^. On the other hand, in specific stress conditions, enhanced glycophagy can also be beneficial for tumor survival^[124,125]^. When tumor cells are faced with hypoxia or nutrient deficiency, activated glycolysis can provide key energy and metabolic intermediates, helping them overcome the stress and maintain proliferation^[126]^. This seemingly opposite effect of “weakened glycolysis promoting tumors” highlights the condition-dependent nature of glycolysis in tumor biology^[19,127]^.

Apart from tumors, glycophagy also exhibits different roles in other diseases: in nonalcoholic fatty liver disease (NAFLD), abnormal glycogen deposition accompanied by fusion disorders of autophagosomes and lysosomes suggests that reduced efficiency of glycophagy may exacerbate hepatocyte lipotoxicity and damage^[128,129]^. In myocardial ischemic injury, the upregulated glycophagy function specifically manages glycogen granules to meet the energy demands of myocardial cells^[66]^. Studies have shown that Asiatic acid (AA) can improve myocardial energy balance by activating glycophagy and mitochondrial autophagy, thereby alleviating ischemic myocardial injury. AA promotes the activity of glycophagy and mitochondrial autophagy through the PI3K/Akt and AMPK signaling pathways^[65]^. In summary, whether glycogen phagocytosis promotes or inhibits tumor growth (or plays a protective role in other diseases) depends on various factors, such as tumor type, genetic background (such as STBD1 mutation status), and microenvironment (such as the supply of nutrients) – thus resolving the initial contradictions in the research results.

#### Prospect

Glycophagy, as an important mechanism for cellular metabolic regulation, has broad research prospects and significant potential for clinical translation. Future research can focus on the mechanism of glycophagy in metabolic diseases, such as diabetic cardiomyopathy and glycogen storage disease, to explore its potential as a therapeutic target. By regulating the activity of key proteins or targeting signaling pathways, new drugs are expected to be developed to improve glycogen metabolism disorders. Moreover, the interaction between glycophagy and other metabolic pathways provides new ideas for energy metabolism remodeling, especially in ischemic myocardial injury and nonalcoholic fatty liver disease. Combining genomic and metabolomic technologies, analyzing the heterogeneity of glycophagy in different disease subtypes, is expected to achieve individualized treatment strategies. At the same time, the potential role of glycophagy in delaying cellular aging and neurodegenerative diseases suggests that it may become a new target for anti-aging and neuroprotection. Through interdisciplinary integration research, combining technologies such as bioengineering and AI-assisted drug design, the screening and validation of glycophagy regulatory molecules are expected to be accelerated, promoting the transformation of basic research to clinical application. In summary, the research on glycophagy is expected to break through the treatment bottlenecks of metabolic diseases and provide innovative solutions for achieving “metabolic homeostasis remodeling.”

## Data Availability

This review is based entirely on publicly available data. All cited studies, databases, and repositories are referenced in the article and can be accessed via the original sources listed in the Re ferences section.
